# Rushed health workforce reform in South Korea: a Kingdon’s multiple streams framework analysis of the 2024 medical school quota expansion

**DOI:** 10.3389/fpubh.2025.1673605

**Published:** 2025-11-11

**Authors:** Yuri Lee, Hyun-Young Shin

**Affiliations:** 1 Department of Health and Medical Information, Myongji College, Seoul, Republic of Korea; 2 Department of Family Medicine, Seoul St. Mary’s Hospital, College of Medicine, The Catholic University of Korea, Seoul, Republic of Korea

**Keywords:** medical education, health policy, workforce planning, Kingdon’s multiple streams, essential care

## Abstract

**Background:**

In 2024, South Korea expanded medical school quotas to address physician shortages in underserved regions and essential specialties. Public concerns emerged over equity and distribution amid political pressure, limited stakeholder participation, and opaque workforce forecasts.

**Objective:**

To explain how political dynamics, stakeholder exclusion, and weak integration of evidence shaped rapid policy change, and to assess implications for workforce planning and essential-care access.

**Methods:**

We conducted a mixed-methods policy analysis using qualitative data from policy documents, legislative records, and media coverage, alongside secondary quantitative data on physician distribution and residency application trends. Thematic coding and triangulation were guided by the three streams of Kingdon’s multiple streams framework: problem, policy, and politics.

**Results:**

The findings reveal that policy urgency was driven by focusing events such as maternal emergencies and pediatric access crises. While numeric expansion was politically favored, workforce projections were inconsistent and failed to address specialty-specific and regional gaps. Political actors prioritized electoral considerations over evidence-based, inclusive reform strategies.

**Conclusion:**

Quota expansion alone is unlikely to resolve disparities. Sustainable reform requires transparent forecasting, targeted incentives, capacity for priority specialties/regions, and participatory governance with multi-stakeholder collaboration.

## Highlights

- A careful and evidence-based approach that aligns problem, policy, and political streams is essential to strengthen essential and regional healthcare, rather than relying on politically driven and hasty quota expansion.
- Increasing admission quotas alone does not ensure sustainability in key specialties or underserved regions; targeted incentives and inclusive governance must accompany expansion.
- Predictive models of physician workforce needs vary widely across countries due to contextual and institutional factors; transparent data sharing and broad stakeholder consensus are critical for effective and equitable policy implementation.

## 1 Background

In South Korea, policymakers have introduced substantial increases in medical school quotas to address chronic physician shortages in critical areas such as emergency medicine, pediatrics, obstetrics, and critical surgical specialties—namely general, thoracic, and neurosurgery (1, 2). The underlying rationale is straightforward: expanding the pool of newly trained physicians, in principle, should strengthen healthcare accessibility in underserved regions and bolster essential services through a trickle-down effect, where an increased supply of doctors would naturally flow to areas of greatest need (3). The policy does not impose mandatory placement into these specialties; instead, it assumes that expanding the total pool of medical graduates will eventually increase the number who voluntarily choose careers in underserved fields and regions. In other words, the reform relies on a trickle-down effect, where higher aggregate supply is expected—though not guaranteed—to ease shortages in essential specialties.

Paradoxically, the quota expansion produced several unintended consequences. Most visibly, it triggered mass resignations among residents and leaves of absence among medical students, who protested what they viewed as a hastily imposed policy (4). Instead of stabilizing the workforce, the rapid increase appeared to intensify fragility, as persistent shortages in core specialties remained and patient caseloads in metropolitan centers continued to grow (5). Analysts note that these paradoxical effects stemmed from the exclusion of professional associations, health administrators, and trainees during policy formulation, which undermined legitimacy and implementation capacity.

Observers increasingly argue that the abrupt expansion was driven more by short-term political imperatives than by balanced, data-driven strategy (6). Comparative research also shows that rushed health reforms in other settings—such as in Latin America and parts of Europe—have produced similar backlashes when frontline conditions and stakeholder input were neglected (7, 8).

Framed through Kingdon’s Multiple Streams Framework (MSF), the Korean case illustrates how the perceived shortage of physicians (problem) converged with a politically expedient solution of numeric expansion (policy), but without adequate deliberation or stakeholder engagement in the politics stream (9, 52). This imbalance underscores the risks of premature policy closure and highlights the need for transparent forecasting, participatory governance, and evidence-based planning that better incorporates those most directly affected by reform (10–12).

Although numerous studies worldwide have examined physician workforce dynamics—ranging from spatial distribution inequalities to future supply–demand forecasts (13–15)—relatively few have tackled how deficient policy procedures can engender the very crises reforms aim to resolve. Existing projections, for instance, often highlight numeric shortfalls without detailing how rushed implementation or constrained dialog might derail otherwise sensible interventions (14, 15). Within the Korean context, most workforce analyses offer quantitative estimates but overlook political expediency or stakeholder marginalization as key factors shaping policy outcomes (12).

Comparative evidence from Europe and South America demonstrates similar tensions between policy ambition and governance constraints. In Poland, for example, a recent descriptive analysis found that admission limits for medical degrees increased by over 90% between 2013 and 2023, including large expansions in private institutions, but these changes often occurred without coherent long-term strategic planning or quality control mechanisms (16). In Spain, “medical deserts”—areas with poor access to physicians—remain a persistent problem despite national shortages, with rural regions lagging behind even as overall physician density increases (17). Brazil’s health system reforms similarly illustrate that while expansion of coverage and workforce programs (e.g., Mais Médicos) aimed at underserved areas, political shifts, funding instability, and weak stakeholder participation undermined sustained impact (18, 19).

With MSF-based studies revealing how policy windows can open following media coverage or public alarm (20, 21), few systematically assess how a lack of robust collaboration and transparent governance can transform policy alignment into large-scale workforce disruptions (5). Thus, a focused application of MSF may illuminate why expansions undertaken with ostensibly beneficial intentions can produce destabilizing sequelae.

The objective of this study is to analyze the unintended consequences of South Korea’s recent expansion of medical school quotas, using Kingdon’s Multiple Streams Framework (MSF) to explore the interaction between the problem, policy, and political streams. Specifically, the study aims to assess how political imperatives, rushed implementation, and a lack of comprehensive stakeholder involvement have shaped the policy outcomes, contributing to workforce instability and exacerbating existing disparities in essential medical services. The scope of the research includes a detailed examination of the physician shortage crisis in critical areas such as pediatrics and emergency medicine, the rapid expansion of medical school admissions, and the political pressures that influenced the policy decision. The study also seeks to highlight the gaps in evidence-based planning and participatory governance that have undermined the long-term effectiveness of the policy. By applying MSF, the research aims to provide insights into how policy decisions, driven by expedient political motivations, may fail to address the root causes of healthcare workforce imbalances and to offer recommendations for more sustainable, inclusive healthcare reforms in South Korea and other high-income countries facing similar challenges.

## 2 Methods

### 2.1 Study design

This study employs a mixed-methods approach, combining qualitative case study analysis and quantitative data analysis, to examine the policy process surrounding the recent debates on expanding medical school quotas in South Korea. The qualitative case study analysis is guided by Kingdon’s MSF, allowing for a detailed exploration of how the problem of physician shortages, the policy of medical school expansion, and political pressures intersect to shape decision-making. Quantitative data analysis is employed to assess trends in medical school admissions, physician distribution, and healthcare utilization, providing contextual insights into the problem stream and policy implications.

### 2.2 Data collection

A range of policy and legislative documents were analyzed, including white papers, policy briefs, official reports, and committee minutes from the Ministry of Health and Welfare (MOHW), the National Assembly Health and Welfare Committee, and other central government agencies such as the Ministry of Education (MOE) and the Health Insurance Review and Assessment Service (HIRA). These documents were selected if they directly addressed physician workforce supply, medical school admissions, or essential-care service shortages; purely descriptive administrative notes and duplicate materials were excluded. This corpus provided insights into the government’s position on the medical school quota debate, proposed policy changes, and the rationale behind these proposals (4, 22).

Media coverage of the policy debates was reviewed to understand how the public discourse surrounding the quota expansion was framed. We purposively sampled coverage from major conservative, progressive, and centrist daily newspapers, as well as medical trade outlets, to capture diverse perspectives. To minimize bias, key events (e.g., maternal transfer cases, pediatric “open-run” phenomena) were cross-checked across ideologically different outlets. The media analysis focused on how physician shortages and healthcare access issues were presented to the public, as well as the political dynamics influencing public opinion (6, 23).

Quantitative data from official sources, including the Korean Statistical Information Service (KOSIS) and HIRA Big Data Open System, were used to analyze trends in medical school admissions, physician distribution across regions, and healthcare utilization patterns. Where official data were incomplete—for example, in specialty-specific residency applications—figures were triangulated from government announcements and corroborating media reports. These data provided essential context for understanding the broader demographic and healthcare workforce trends that influence the policy process (12).

A review of academic literature was conducted to gather research on healthcare workforce issues, medical education reforms, and the regional distribution of healthcare professionals. Searches were carried out in PubMed, Scopus, and RISS (Research Information Sharing Service) using keywords such as “physician workforce,” “medical education reform,” and “regional distribution” for the period 2010–2024. Peer-reviewed studies were included if they examined physician supply, demand, or distribution; commentaries and non-academic reports were excluded. Relevant studies were drawn from peer-reviewed journals discussing physician supply and demand, and the challenges associated with medical workforce distribution in South Korea (5, 7).

### 2.3 Data analysis

Thematic coding was applied to policy documents, media articles, and other secondary data sources to identify recurring themes related to political motivations, stakeholder influence, and the framing of healthcare issues. The coding process was guided by Kingdon’s MSF, focusing on the three streams—problem, policy, and politics. Initial codes were developed inductively after repeated readings of the data, and a codebook was refined iteratively to capture both predefined MSF categories and emergent themes. Coding was carried out by two researchers who compared results and resolved discrepancies through discussion to ensure consistency. Media reports and policy documents were treated with equal weight at the coding stage, but triangulation prioritized official policy documents where discrepancies arose, with media used primarily to capture framing dynamics (53).

Triangulation was employed to cross-check data across multiple sources, including policy documents, media coverage, and academic literature. This helped to ensure that the findings were consistent and reliable. For instance, the data on political pressures were compared with media portrayals and government reports, providing a more comprehensive understanding of the political stream (9).

A detailed analysis of the political stream was carried out by examining the influence of electoral cycles, regional political pressures, and political lobbying by medical associations. This analysis mapped out the political landscape and identified the key political figures involved in the decision-making process, as well as their stances on the quota expansion (7, 11).

Quantitative data from KOSIS and other government sources were analyzed to identify trends in medical school admissions, physician-to-population ratios, and healthcare utilization across regions. Descriptive statistics were used to calculate the physician-to-population ratio for different regions (Seoul vs. non-metropolitan areas) and to assess the correlation between medical school quotas and the geographic distribution of healthcare professionals. The analysis also involved examining the regional disparities in physician availability and assessing the relationship between medical school quotas and physician shortages in underserved areas (24).

Scenario simulations were used to project the impact of maintaining current medical school quotas versus expanding them. Two scenarios were modeled: (1) a status quo scenario maintaining the current quota of 3,058 seats, and (2) an expansion scenario reflecting the government’s proposed increase to 5,053 seats beginning in 2025. The simulations incorporated demographic variables such as population aging, annual birth counts, and the projected size of the 18-year-old cohort, as reported by KOSIS. Assumptions regarding physician migration between regions and specialty choices were based on HIRA data and previously published workforce studies.

**Figure 1:**
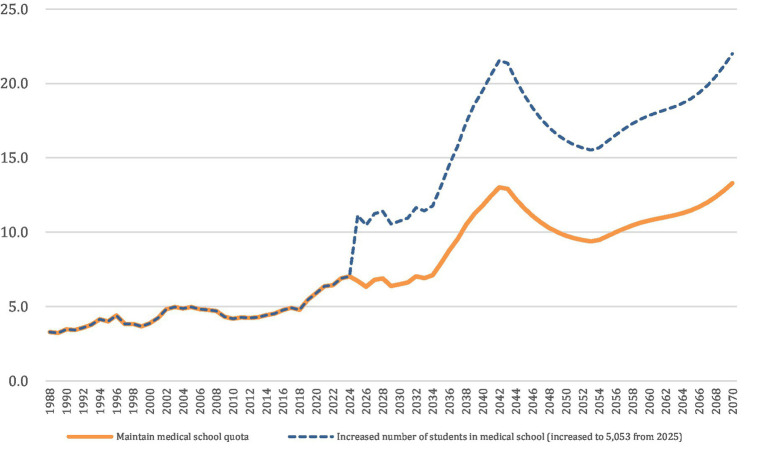
Estimated medical school quota ratio compared to the number of students expected to enter college (age 18). This figure utilizes KOSIS data on annual birth counts and projections for the 18-year-old population to estimate the number of prospective college entrants. The medical school quota data are based on government and media announcements, assuming an increase to 5,053 seats from 2025 onward. “Maintain medical school quota” reflects the status quo, while “Increased number of students in medical school (5,053 from 2025)” illustrates an expansion scenario.

The model calculated the ratio of available medical school seats to the number of prospective college entrants (age 18) to assess whether expansion aligned with long-term demographic capacity. For example, Figure 1 utilizes KOSIS data on annual birth counts and projections for the 18-year-old population to estimate the number of prospective college entrants. The medical school quota data are derived from government and media announcements, assuming an increase to 5,053 seats from 2025 onward. “Maintain medical school quota” reflects the status quo, while “Increased number of students in medical school (5,053 from 2025)” illustrates an expansion scenario. Results were compared with qualitative findings to assess whether quota expansion alone could resolve shortages in critical specialties.

Assumptions underlying the model included stable graduation rates, constant physician migration trends, and no major shocks to specialty preferences during the projection period. Limitations should also be acknowledged: the simulations did not capture potential policy feedback effects, evolving retirement patterns, or changing patient demand beyond demographic projections. These boundaries are noted to ensure transparency and to clarify that the projections provide indicative rather than deterministic outcomes.

### 2.4 Application of Kingdon’s multiple streams framework

Kingdon’s Multiple Streams Framework (MSF) was employed to analyze the policy process and examine how the three streams—problem, policy, and politics—converged to shape the policy outcome. MSF provided a useful framework for understanding how the policy window opened for the expansion of medical school quotas, and how political pressures, stakeholder advocacy, and framing of healthcare issues interacted during the policy debate.

With respect to the problem stream, the study explored how the issue of physician shortages, particularly in essential medical fields, was framed by policymakers and media outlets. It examined how public concern over healthcare access and physician distribution helped elevate the issue onto the policy agenda (7). Regarding policy stream, the analysis focused on the policy proposals related to increasing medical school quotas, evaluating whether these proposals were grounded in evidence of actual healthcare needs or were primarily driven by political imperatives. The study also examined the role of policy entrepreneurs in advocating for or against the policy changes (5).

For the political stream, the analysis was expanded to systematically examine how electoral cycles, lobbying, and regional pressures influenced the decision-making process. Electoral timing was coded using the 2024 general election as a temporal marker, allowing us to trace how proximity to elections accelerated government announcements and shaped the framing of physician shortages as an urgent issue. Lobbying was analyzed through the frequency and framing of official statements, press releases, and National Assembly testimonies by the Korean Medical Association and other professional groups, which revealed the extent of organized resistance to the reform. Regional pressures were assessed by coding references in policy documents and media reports to persistent shortages in pediatrics, obstetrics, and emergency medicine in non-metropolitan provinces. These political factors were triangulated with legislative records and media coverage to ensure consistency and reduce interpretive bias (11, 25).

This stream-based approach made it possible to capture how political pressures shaped the choice of expanding medical school quotas as the preferred policy solution, while also highlighting the limited deliberation and stakeholder inclusion that accompanied the rapid policy shift.

### 2.5 Ethical considerations

As this study primarily utilizes secondary data from publicly available documents, media coverage, and official reports, ethical approval and informed consent were not required. However, all data sources were appropriately cited, and confidentiality was maintained by ensuring that no personally identifiable information was included in the study (Table 1).

**Table 1:** Applying Kingdon’s multiple streams framework to the medical school quota expansion policy.

Stream	Key concepts	Observations in the context of medical school quota expansion
Problem stream	- The process by which certain issues receive attention and are identified as problems requiring action - Driven by indicators, focusing events, and media/public sentiment	- Persistent shortages in essential medical specialties (pediatrics, obstetrics, emergency medicine) and urban–rural disparities gained visibility through high-profile “focusing events,” such as “open-run” pediatric clinics and emergency department “roundabouts.” - Media reports highlighted cases where patients struggled to access urgent or specialized care, which in turn fueled public pressure for immediate solutions.
Policy stream	- The “policy primeval soup,” where experts, interest groups, and policymakers generate and refine potential solutions - Ideas compete to become viable policy alternatives - Technical feasibility and alignment with values often shape which solutions gain traction	- Numerical expansion (increasing medical school seats) emerged as a simple, quantifiable fix to the perceived physician shortage. - Competing policy proposals (e.g., targeted incentives for essential specialties, regional bonding programs) received less political traction, partly due to their complexity and longer implementation timelines. - Ambiguity in physician workforce projection data (different methodologies or indicators) complicated consensus on how many doctors are truly needed and in which specialties/locations.
Politics stream	- Political climate, party agendas, elections, and interest group pressures determine what is politically feasible - Shifts in public opinion or leadership can rapidly open or close opportunities for policy adoption	- Political urgency to demonstrate action (often tied to electoral considerations) accelerated the push for expanding medical school quotas. - High-level political figures framed “increasing the quota” as an immediate, visible solution, overshadowing the necessity for deeper structural reforms. - Lobbying by professional associations, medical schools, and other stakeholders influenced the debate, sometimes resulting in compromises that neglected comprehensive regional or specialty-based strategies.

## 3 Results

### 3.1 Problem stream

Public alarm over essential healthcare gaps intensified when a pregnant individual in Gangwon Province had to be airlifted 200 km to Seoul in 2023 due to the absence of local obstetric facilities. Media coverage of such critical incidents drew attention to the severe lack of essential specialties—defined in the Korean context as areas directly related to life-threatening conditions (e.g., emergency medicine, obstetrics, pediatrics, and critical surgical fields) that require immediate intervention or state support when market viability is low (1). These shortages have direct implications for health outcomes: limited obstetric capacity threatens timely maternal emergency care, insufficient pediatric coverage leads to “open-run” queues and restricted outpatient access, and inadequate emergency medicine specialists contribute to overcrowded ERs and repeated transfers of critically ill patients. Such patterns mirror similar patterns in other settings where dramatic focusing events prompt sudden shifts in policy priorities (7, 20). During the COVID-19 pandemic, the significance of a strong public healthcare system and the depth of healthcare workforce shortages became even more evident, further amplifying calls for structural reforms.

**Figure 2:**
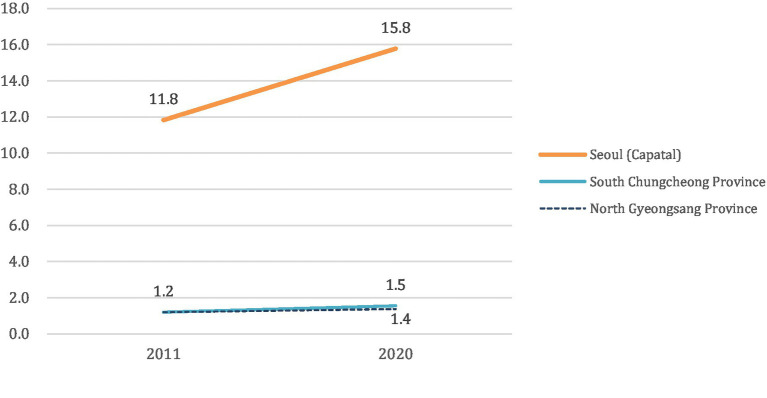
Differences in the ratio of doctors working at medical institutions by region per 1,000 population (weighted by medical use) between 2011 and 2020. This figure was generated by (1) extracting 2011 and 2020 population data for Seoul, South Chungcheong, and North Gyeongsang from KOSIS (Korean Statistical Information Service https://kosis.kr/index/index.do) by age group, and (2) deriving age-specific utilization weights (daily care utilization, per capita medical expenditure) from the HIRA Big Data Open System (Health Insurance Review & Assessment Service https://opendata.hira.or.kr/home.do). We then multiplied each age-group population by its respective weight to calculate a “weighted population” (∑(age-group population × utilization weight)) for each region. The number of practicing physicians in 2011 and 2020 was obtained from administrative statistics (e.g., Ministry of Health and Welfare, Korean Medical Association), and divided by the weighted population to yield physicians per 1,000 weighted persons. This approach accounts for age-specific healthcare utilization, thereby offering a more refined view of regional healthcare accessibility than a simple ratio of physicians to total population.

Data provided by civic organizations and government sources further revealed extensive understaffing across public healthcare institutions, including local medical centers and university-affiliated hospitals. As shown in Figure 2, based on KOSIS and HIRA data, the number of physicians per 1,000 population in the capital region (Seoul) increased from 11.8 to 15.8 between 2011 and 2020, whereas South Chungcheong and North Gyeongsang provinces remained below 7.0 during the same period, confirming persistent disparities already highlighted in our Methods section. These findings illustrate how uneven geographic distribution persists, leaving rural regions acutely vulnerable. Such visible discrepancies align with Kingdon’s conceptualization that urgent, high-profile shortfalls heighten public anxiety, thus amplifying the salience of these issues within the problem stream (21, 26).

Attempts to resolve these disparities through more appealing compensation schemes have had limited success in stabilizing critical services in underserved fields. Persisting structural barriers—such as restricted professional development opportunities and inadequate clinical infrastructure—are likely to be pivotal constraints. A growing body of multiple-streams research suggests that policy measures addressing superficial, rather than systemic, factors often prove insufficient for alleviating the broader crisis.

Mounting public concern has thus elevated the urgency of essential healthcare shortages to a national debate, spurring calls for wider, evidence-based solutions that go beyond expanding medical school admissions. As the problem stream intensifies, policymakers are under greater pressure to explore integrated reforms, echoing prior findings that once an issue is framed as a looming crisis, it gains the momentum necessary to prompt more robust and inclusive policy responses (11, 27).

### 3.2 Policy stream

Expansion of medical school quotas emerged as a prominent policy proposal to address the conspicuous shortfalls in essential specialties (4, 7). The conceptual scope and definition of essential specialties commonly accepted in South Korea encompass areas directly related to life-threatening conditions (such as critical care, severe trauma, and cardiovascular and cerebrovascular diseases). It is based on the timeliness of diseases requiring “immediate and appropriate action,” including emergency treatment, emergency surgery, and emergency childbirth. It also includes medical services necessary for the public that are clinically beneficial but lack market viability and thus require state support and development (28). Policymakers gravitated toward simple, numeric indicators—most notably, the physician-to-population ratio—when articulating the need for large-scale growth in medical education programs (7, 29). However, multiple-streams scholarship warns that such easily quantifiable measures may obscure deeper, more persistent issues (11, 26).

**Figure 3:**
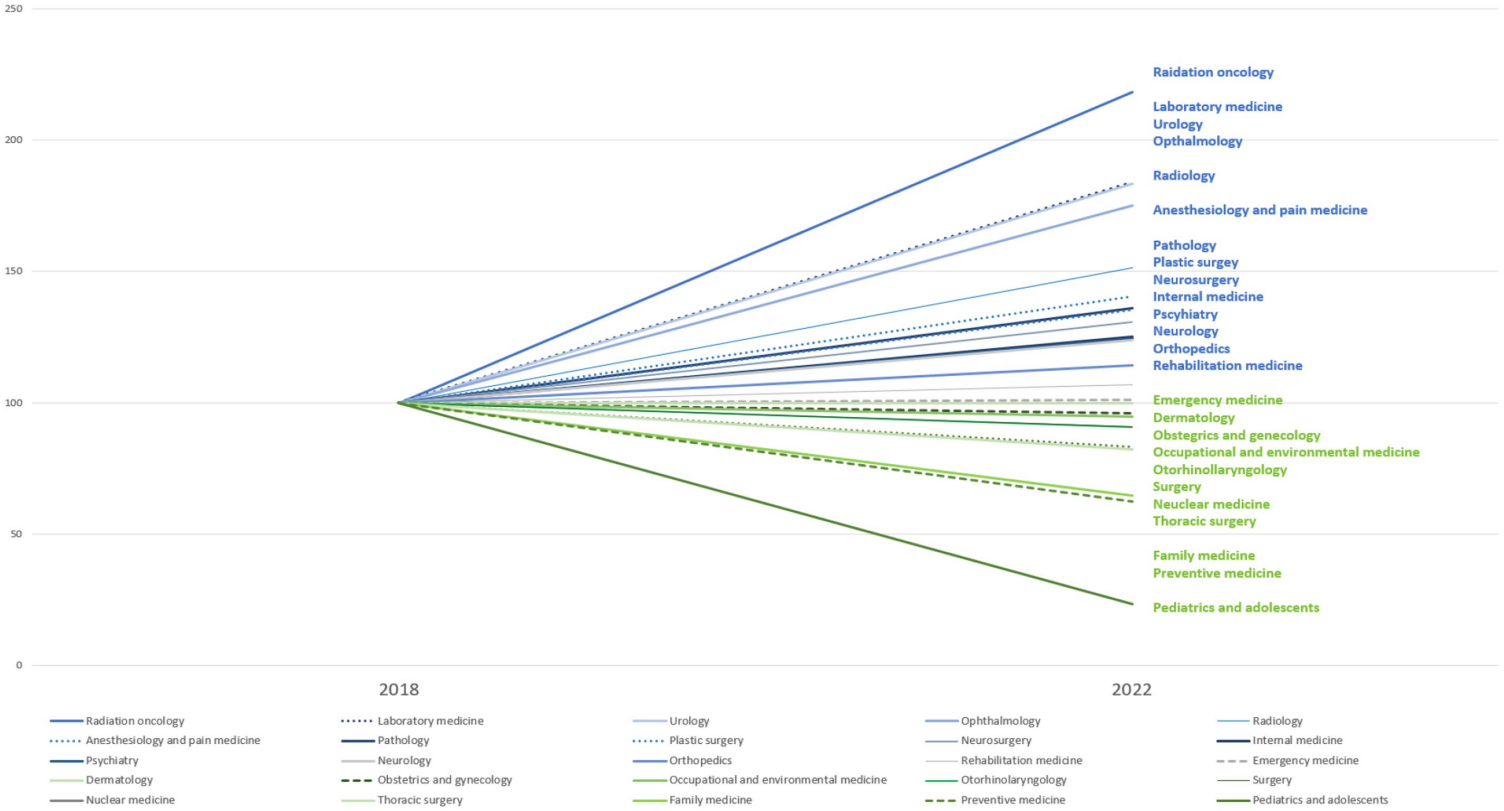
Change in total number of residency applicants by medical specialties between 2018 and 2022. Residency applicant data from 2018 to 2022 were compiled from publicly disclosed figures in major media outlets, which reported official announcements regarding specialty-specific application trends. This approach was used because no single consolidated dataset was available at the time.

Figure 3 exemplifies one central problem in the policy stream: the decline in residency applicants for essential-care specialties from 2018 to 2022. Obstetrics residency applicants dropped from 155 to 119 between 2018 and 2022, while pediatrics declined from 381 in 2011 to just 97 in 2022, representing a 75% decrease over a decade. Although additional medical students are entering the system overall, the downward trend for certain specialties emphasizes that numeric expansion alone does not funnel physicians into high-need areas (5). This mismatch has tangible effects on service delivery, as reduced recruitment into obstetrics and pediatrics undermines maternal and child health services, while persistent shortages in emergency medicine continue to compromise timely and equitable emergency care. The persistence of “open-run” pediatric clinics and recurring emergency department “roundabouts,” despite nearly two decades of frozen admission quotas, demonstrates that simply expanding medical school seats without addressing structural factors such as training capacity, incentives, and working conditions is unlikely to resolve shortages (1, 30, 31).

Policymakers have frequently invoked future workforce projections to justify aggressive expansions of medical school seats. Yet these projections often rely on data that capture only broad, aggregate patterns of physician supply and demand [Song, 2023; (4)]. Figure 1 underscores this point by illustrating how the ratio of medical school quotas to prospective college entrants can rise significantly without guaranteeing that the additional graduates will pursue or remain in essential specialties. The so-called “streetlight effect” materializes when decision-makers concentrate on readily available metrics—like absolute physician numbers—while neglecting structural factors such as training capacity, payment structures, and working conditions in essential fields [Song, 2023; (32)].

The policy stream is characterized by a strong push for an enrollment-centered solution to perceived physician shortages, albeit with mounting evidence that this approach overlooks vital nuances. Although various stakeholders—including medical associations, hospital administrators, and policy researchers—have recommended more finely tuned strategies (8, 10), the public debate often reverts to numerical expansions as a visibly decisive political solution. This limited range of policy alternatives highlights a core shortcoming in the policy stream: the absence of robust, multipronged reforms that address the root causes of workforce maldistribution in essential medical services (4, 15).

### 3.3 Political stream

Political motivations have played a dominant role in shaping the discourse surrounding the expansion of medical school quotas in South Korea. These motivations often prioritize immediate political gains over a more systematic, evidence-based approach to resolving healthcare workforce issues. Although various well-intentioned measures to reduce regional disparities and address workforce shortages were proposed, short-term electoral objectives diminished the policy’s long-term effectiveness (33). This strategy is closely tied to political objectives, with arguments for an expanded physician workforce framed as a solution to regional inequities and a means of addressing electoral promises regarding local development (7). The timing of the (12) general election further amplified political incentives to frame quota expansion as an urgent solution to healthcare disparities, aligning the reform agenda with electoral strategies. Such political framing underscores Kingdon’s assertion that policy decisions are often shaped by what is politically feasible at the time, which can sometimes override the importance of a long-term, evidence-based strategy (52).

However, the political pressures surrounding this policy have led to concerns over the policy’s long-term sustainability and effectiveness. Proposals to increase medical school quotas—while politically expedient—fail to address the root causes of workforce shortages in critical specialties like obstetrics and pediatrics. As highlighted by recent critiques, the political push for quota expansion is often framed as a quick and simple solution, sidelining the deeper structural issues related to workforce distribution, specialty preference, and retention (5). For instance, although there is a strong political drive to increase the number of medical students, the actual distribution of these new physicians across regions and specialties remains uncertain. The increasing emphasis on non-metropolitan regions may be politically motivated, as it aligns with electoral strategies aimed at garnering support from rural voters, but it does not necessarily address the specific healthcare needs of underserved areas, such as specialty training in pediatrics or emergency medicine (7).

Another key feature of the political stream is the substantial influence wielded by specific political stakeholders and academic groups, which has led to an overrepresentation of their interests in the policy discussion. Medical professionals, including the Korean Medical Association, have been highly vocal in shaping the policy agenda, often through public statements, press conferences, and testimonies in the National Assembly, and frequently advocating for policies that reflect their interests, which can include opposing reforms that threaten their perceived professional autonomy (10). However, the perspectives of patients, civic organizations, and frontline providers—voices often reflected in media reports and secondary sources—were largely excluded from the decision-making process. The absence of these groups not only deepens participatory deficits but also undermines the legitimacy and responsiveness of the reform, since those most directly affected by the shortages were not adequately represented. This concentration of influence has been criticized for sidelining the perspectives of local residents and frontline healthcare workers, who are arguably the most directly impacted by these policies. The absence of a more inclusive decision-making process has undermined the legitimacy of the reform, resulting in public disillusionment and protests from various healthcare groups (32). For example, the exclusion of regional voices from policy deliberations has contributed to a lack of trust in the government’s ability to manage healthcare reform effectively, as the voices of those who would benefit from or be impacted by the changes are not adequately heard (4).

Moreover, the political process has been criticized for bypassing scientific rigor in favor of quick, politically advantageous decisions. This trend undermines the long-term effectiveness of the policy by neglecting to incorporate detailed demographic analyses and projections of healthcare needs (34). A prime example of this was the lack of a systematic, independent process to project the future demand for healthcare professionals, with many experts arguing that the current approach to workforce planning is based more on political agendas than on scientific data. The absence of a well-structured governance framework for physician workforce forecasting, such as an independent support committee to oversee medical workforce projections, has led to a situation where the policy is shaped more by political considerations than by evidence-based solutions (35). Similar dynamics have also been observed internationally, for example in Spain and Brazil, where electoral cycles accelerated health workforce reforms but weakened their long-term sustainability (8). As a result, the policy risks being short-sighted, failing to adequately address the complexities of the healthcare system, and leading to future challenges in achieving a balanced and sustainable medical workforce (8, 27).

## 4 Discussion

This study provides a comprehensive examination of the policy process surrounding the expansion of medical school quotas in South Korea, applying Kingdon’s MSF to understand how political, policy, and problem streams intersected to drive the debate. Beyond applying MSF descriptively, the study also engages with its theoretical boundaries by examining how electoral imperatives and forecasting uncertainty interact to shape policy trajectories, an aspect not fully anticipated in the original framework. The findings suggest that while the medical school quota expansion addresses an urgent issue of physician shortages, particularly in essential specialties such as obstetrics, pediatrics, and emergency medicine, several challenges remain regarding the effectiveness of this solution.

The problem of physician shortages, particularly in essential medical specialties, was exacerbated by high-profile events such as the “open-run” phenomenon in pediatric clinics and emergency room “roundabouts” (22, 36). These focusing events elevated public awareness and added significant pressure on policymakers to respond, aligning with Kingdon’s assertion that crises can catalyze policy change (52). The public’s frustration with delayed or unavailable care, coupled with media coverage, prompted swift political action. However, the policy response, namely the increase in medical school quotas, has sparked considerable debate regarding its potential to resolve the core issues of healthcare access and physician distribution (4, 12).

As shown in Figure 3, the declining number of residency applicants in critical specialties such as obstetrics and pediatrics from 2018 to 2022 further indicates that simply increasing the number of medical students does not necessarily address the need for specialized healthcare professionals in underserved areas. Stakeholders from the medical community, including professional associations and academic bodies, have questioned whether an immediate expansion of quotas without complementary policies, such as region-specific scholarships or mandatory service in rural areas, will effectively alleviate healthcare gaps (7, 12). This reflects the ongoing tension in the policy stream, where a focus on increasing physician numbers overshadows deeper structural challenges in healthcare distribution (5).

A significant challenge in the policy process lies in inherent ambiguity of physician workforce projections, as different variables, indicators, and modeling assumptions often yield markedly divergent forecasts ranging from massive shortages to near surpluses (1, 12). Our analysis suggests that this forecasting ambiguity became a political resource when combined with electoral imperatives, providing justification for rapid policy change. This dynamic highlights a blind spot in MSF, where technical uncertainty itself can function as a driver of political feasibility. Pasha et al. (37) similarly underscore that such complexities have prompted calls for a unified, transparent national framework to guide physician workforce policy, a challenge highly relevant to the Korean context. International evidence also underscores these difficulties: Sutton et al. (38), in a scoping review of strategic workforce planning in health and social care, found that approaches differ widely across contexts, complicating direct comparisons but offering valuable lessons for cases such as Korea. This forecasting uncertainty not only creates confusion among policymakers but also undermines public trust in the resulting decisions. Consequently, ensuring a transparent, collaborative approach to workforce projection—where data sources, analytic methods, and assumptions are openly shared and subjected to broad stakeholder review—is critical. Such procedural transparency can help build social consensus around the most appropriate policy responses, including decisions on expanding medical school quotas. A recent systematic review by Bosak et al. (39) further highlights that workforce planning models differ substantially in their approaches and components, reinforcing the difficulty of producing consistent and comparable forecasts. Without this collective agreement and clarity in forecasting, even seemingly essential measures, such as increasing admissions, risk failing to resolve specialty imbalances or regional disparities in the physician workforce (4, 22).

Future research should address this issue by developing a more unified framework for workforce projections, one that incorporates shifting demographic and technological factors to provide a more accurate forecast. A formalized structure, such as the proposed Health Workforce Supply and Demand Projection Committee (35), could systematically collect data and refine forecast models to guide future policymaking in a more evidence-driven manner. Recent studies in Korea have also emphasized the need for a Korean-style projection model that integrates population aging, regional disparities, and evolving primary care demands (40–42). Such models stress that quota expansion must be complemented by targeted incentives, governance reform, and new delivery arrangements if it is to achieve sustainability. At the global level, Correia et al. (43) argue that turning the current workforce crisis into effective action requires institutionalizing evidence-based policymaking and strengthening governance mechanisms to translate forecasts into practice. Internationally, OECD (44) and WHO (45) reports have also highlighted forecasting uncertainty and regional maldistribution as global challenges, suggesting that Korea’s experience is part of a wider international struggle to align workforce policies with population health needs (44, 45). In line with this, Lev et al. (46) show that even among OECD countries, strategic workforce planning must contend with persistent healthcare inequalities, as illustrated in their comparative analysis of Israel and peer nations.

The political stream has been a dominant force in shaping the current debate on medical school quotas. It is clear that electoral cycles and political imperatives have played a significant role in pushing this policy forward. Our findings also indicate that electoral imperatives interacted with forecasting uncertainty in ways that MSF does not fully anticipate, revealing potential blind spots in the framework. While the problem and policy streams converged, the politics stream accelerated closure of the policy window, raising the question of whether this case represents partial stream convergence or premature closure. This suggests that MSF could be extended to account for how technical uncertainty, when combined with electoral timing, can drive premature decisions and shape policy trajectories (11). Polls show that while the public generally supports the expansion of medical school quotas, many prefer a more nuanced approach that considers regional disparities and specialized needs (23, 47).

Politicians and policymakers are clearly sensitive to electoral pressures, as evidenced by the inclusion of regional quotas and increased representation for rural areas in the proposals (22). However, critics argue that these political motivations may undermine the long-term sustainability of the policy, particularly if it does not address the broader structural challenges in healthcare delivery, such as labor conditions, liability, and financial sustainability (48). The study found that the focus on political feasibility, rather than long-term planning and evidence-based solutions, may lead to unintended consequences that hinder the policy’s ability to resolve the healthcare disparities it aims to address.

While the expansion of medical school quotas is seen as a potential solution to the physician shortage, several structural barriers could undermine its effectiveness. One major concern is the quality of training and the geographic distribution of new physicians. Simply increasing the number of medical students does not guarantee high-quality training or an equitable distribution of physicians across regions (3, 4). Historically, newly trained physicians have gravitated toward urban centers and high-paying specialties, leaving rural areas and essential fields like pediatrics, obstetrics, and emergency medicine understaffed (48, 49). Without targeted policies such as mandatory service in underserved areas or enhanced training programs for critical specialties, medical school expansions may not significantly improve access to care in high-need areas (11).

Moreover, the ambiguous definition of essential medical care continues to hinder the effectiveness of policy planning (2, 28). The lack of a clear, nationally accepted definition of what constitutes “essential” care has created confusion and inefficiencies in resource allocation and healthcare service delivery. Comparative research also indicates that countries such as the Netherlands and the UK have advanced by clearly defining essential services and linking them to workforce planning mechanisms (50). Earlier analyses from Korea Institute for Health and Social Affairs (51) similarly examined the UK and Australian workforce planning models, highlighting how long-term quota adjustments were tied to regional service delivery and quality assurance. More recent international studies also emphasize that unclear definitions of essential care undermine policy effectiveness by obscuring links to health outcomes, such as timely maternal care, equitable pediatric access, and reliable emergency coverage (38, 43). Drawing on these international experiences may provide valuable insights for Korea’s future reforms. Establishing a cohesive and widely accepted definition of essential medical services would therefore be a crucial step not only in streamlining policy development but also in ensuring measurable improvements in healthcare access and quality.

While this study offers valuable insights into the policy process, it has several limitations. First, the study relies heavily on secondary data sources, including policy documents and media coverage, which may not fully capture the nuances of behind-the-scenes political negotiations. These sources, while rich in context, may not always provide a complete picture of the policymaking process or the informal discussions that influence decision-making (9). Additionally, the study is limited to the South Korean context, and while the findings are relevant to South Korea’s healthcare policy, they may not be fully generalizable to other countries with different political and healthcare systems. Moreover, because the research relied primarily on secondary sources, the perspectives of patients, civic groups, and frontline providers could not be directly incorporated, which represents a further limitation of the study.

Future studies should expand by conducting comparative case analyses, for example examining physician workforce reforms in OECD countries, to identify both transferable strategies and Korea-specific challenges. In addition, future research should incorporate primary data from patients, civic organizations, and frontline healthcare providers to more fully capture stakeholder perspectives and to provide a richer understanding of participatory deficits in workforce policymaking. In this sense, Korea’s case provides a useful lens for other high-income countries facing similar demographic transitions and essential-care shortages, demonstrating how political imperatives, forecasting uncertainty, and structural barriers can converge to shape health workforce reform.

## 5 Conclusion

This study highlights the complexities of policy formulation in the context of medical school quota expansion in South Korea. Through the lens of Kingdon’s multiple streams framework, it reveals how political pressures, stakeholder influence, and data-driven policy solutions interact to shape healthcare workforce policy. Beyond descriptive application, the Korean case illuminates theoretical blind spots in MSF by demonstrating how electoral imperatives and forecasting uncertainty interacted to accelerate decision-making. This raises the question of whether the reform represents partial stream convergence or premature closure of the policy window, suggesting that MSF should be extended to better account for the role of technical ambiguity in policy processes.

In practical terms, the findings suggest that while expanding medical school quotas may address some of the immediate physician shortages, a more comprehensive, evidence-based approach is required. Complementary policies to improve geographic distribution, strengthen retention, and ensure training quality are essential for sustainable reform.

Taken together, the study contributes both theoretically, by advancing MSF as a framework capable of explaining how uncertainty and electoral timing combine to shape reform trajectories, and practically, by highlighting policy directions that can enhance equity and resilience in South Korea’s healthcare system. By engaging multiple stakeholders and grounding decisions in rigorous, data-driven projections, South Korea’s healthcare system can better meet the demands of a changing demographic and ensure equitable access to essential medical services.

### 5.1 Reflexivity statement

In conducting this research, the authors collaborated across multiple disciplines (health policy, public health, and clinical medicine) and engaged in critical reflection regarding regional and specialty imbalances in the physician workforce. Both authors represent different career stages, with Dr. Lee and Dr. Shin sharing a commitment to equitable healthcare policies.

## Data Availability

The datasets presented in this study can be found in online repositories. The names of the repository/repositories and accession number(s) can be found at: https://kosis.kr/index/index.do.
