# Supplementary material for: Rushed health workforce reform in South Korea: a Kingdon’s multiple streams framework analysis of the 2024 medical school quota expansion
Source: Front Public Health. 2025 Nov 11;13:1673605. doi: 10.3389/fpubh.2025.1673605 (PMC12644070; doi:10.3389/fpubh.2025.1673605)
Supplement: Supplementary file 1 [file Table_1.DOCX]

Table 1. Applying Kingdon’s Multiple Streams Framework to the Medical School Quota Expansion Policy

| **Stream** | **Key Concepts** | **Observations in the Context of Medical School Quota Expansion** |
| --- | --- | --- |
| **Problem Stream** | - The process by which certain issues receive attention and are identified as problems requiring action  - Driven by indicators, focusing events, and media/public sentiment | - Persistent shortages in essential medical specialties (pediatrics, obstetrics, emergency medicine) and urban-rural disparities gained visibility through high-profile “focusing events,” such as “open-run” pediatric clinics and emergency department “roundabouts.”  - Media reports highlighted cases where patients struggled to access urgent or specialized care, which in turn fueled public pressure for immediate solutions. |
| **Policy Stream** | - The “policy primeval soup,” where experts, interest groups, and policymakers generate and refine potential solutions  - Ideas compete to become viable policy alternatives  - Technical feasibility and alignment with values often shape which solutions gain traction | - Numerical expansion (increasing medical school seats) emerged as a simple, quantifiable fix to the perceived physician shortage.  - Competing policy proposals (e.g., targeted incentives for essential specialties, regional bonding programs) received less political traction, partly due to their complexity and longer implementation timelines.  - Ambiguity in physician workforce projection data (different methodologies or indicators) complicated consensus on how many doctors are truly needed and in which specialties/locations. |
| **Politics Stream** | - Political climate, party agendas, elections, and interest group pressures determine what is politically feasible  - Shifts in public opinion or leadership can rapidly open or close opportunities for policy adoption | - Political urgency to demonstrate action (often tied to electoral considerations) accelerated the push for expanding medical school quotas.  - High-level political figures framed “increasing the quota” as an immediate, visible solution, overshadowing the necessity for deeper structural reforms.  - Lobbying by professional associations, medical schools, and other stakeholders influenced the debate, sometimes resulting in compromises that neglected comprehensive regional or specialty-based strategies. |
