# Supplementary material for: Rushed health workforce reform in South Korea: a Kingdon’s multiple streams framework analysis of the 2024 medical school quota expansion
Source: Front Public Health. 2025 Nov 11;13:1673605. doi: 10.3389/fpubh.2025.1673605 (PMC12644070; doi:10.3389/fpubh.2025.1673605)
Supplement: Supplementary file 2 [file Table_2.DOCX]

Figure 1. Differences in the ratio of doctors working at medical institutions by region per 1,000 population (weighted by medical use) between 2011 and 2020

**Note**: This figure was generated by (1) extracting 2011 and 2020 population data for Seoul, South Chungcheong, and North Gyeongsang from **KOSIS** (Korean Statistical Information Service <https://kosis.kr/index/index.do>) by age group, and (2) deriving age-specific utilization weights (daily care utilization, per capita medical expenditure) from the HIRA Big Data Open System (Health Insurance Review & Assessment Service <https://opendata.hira.or.kr/home.do>). We then multiplied each age-group population by its respective weight to calculate a “weighted population” (∑(age-group population × utilization weight)) for each region. The number of practicing physicians in 2011 and 2020 was obtained from administrative statistics (e.g., Ministry of Health and Welfare, Korean Medical Association), and divided by the weighted population to yield physicians per 1,000 weighted persons. This approach accounts for age-specific healthcare utilization, thereby offering a more refined view of regional healthcare accessibility than a simple ratio of physicians to total population.

Figure 2. Change in total number of residency applicant change ratio by medical specialties between 2018 and 2022


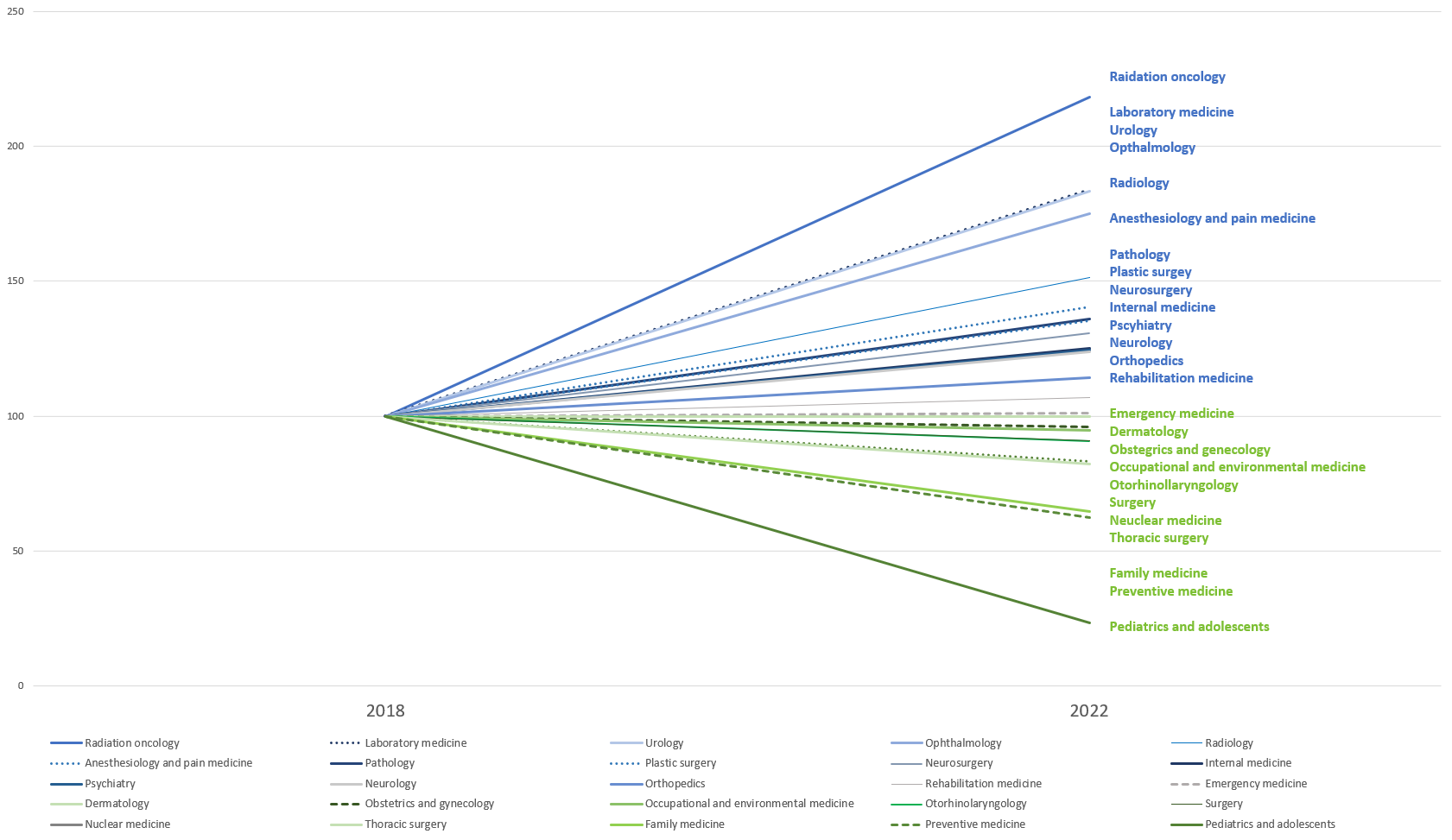


**Note:** Residency applicant data from 2018 to 2022 were compiled from publicly disclosed figures in major media outlets, which reported official announcements regarding specialty-specific application trends. This approach was used because no single consolidated dataset was available at the time.

Figure 3. Estimated medical school quota ratio compared to the number of students expected to enter college (age 18)

**Note**: This figure utilizes KOSIS data on annual birth counts and projections for the 18-year-old population to estimate the number of prospective college entrants. The medical school quota data are based on government and media announcements, assuming an increase to 5,053 seats from 2025 onward. “Maintain medical school quota” reflects the status quo, while “Increased number of students in medical school (5,053 from 2025)” illustrates an expansion scenario.
